# Chance Constrained Input Relaxation to Congestion in Stochastic DEA. An Application to Iranian Hospitals

**DOI:** 10.5539/gjhs.v7n4p151

**Published:** 2014-12-31

**Authors:** Hooshang Kheirollahi, Behzad Karami Matin, Mohammad Mahboubi, Mehdi Mirzaei Alavijeh

**Affiliations:** 1 Islamic Azad University, Sanandaj, Iran; 2 Research Center for Environmental Determinants of Health, Kermanshah University of Medical Sciences, Kermanshah, Iran; 3 Abadan School of Medical Sciences, Abadan, Iran; 4 Social Determinants of Health Research Center, Yasuj University of Medical Sciences, Yasuj, Iran

**Keywords:** stochastic data envelopment analysis, input relaxation, efficiency, hospital

## Abstract

This article developed an approached model of congestion, based on relaxed combination of inputs, in stochastic data envelopment analysis (SDEA) with chance constrained programming approaches. Classic data envelopment analysis models with deterministic data have been used by many authors to identify congestion and estimate its levels; however, data envelopment analysis with stochastic data were rarely used to identify congestion. This article used chance constrained programming approaches to replace stochastic models with ‘‘deterministic equivalents”. This substitution leads us to non-linear problems that should be solved. Finally, the proposed method based on relaxed combination of inputs was used to identify congestion input in six Iranian hospital with one input and two outputs in the period of 2009 to 2012.

## 1. Introduction

Data envelopment analysis (DEA) as a non-parametric technique has been widely used to measure the relative efficiency of a set of similar decision making units (DMUs) which was introduced in the year 1963 by Charnes and Cooper (Charnes & Cooper, 1963). The first model in DEA was called CCR, to determine the efficiency of US public school education. Banker, Charnes and Cooper developed a variable returns to scale that was called BCC model (Banker, 1984) in 1984. Identifying and estimating congestion as the severe form of inefficiency plays an important role in evaluating production. Congestion is present in the performance of decision making unit (DMU) when reductions in one or more inputs are associated with increases in one or more outputs—without worsening any other input or outputs. More precisely, congestion is evidenced when the attainment of maximal output requires a reduction in one or more of the input amounts used; Cooper et al. introduced an approach model to congestion in DEA in 2002 (Cooper et al., 2002).

Kirigia et al. introduced an input relaxation model in DEA that allows increase of inputs to improve outputs for units which are inefficient (Kirigia, et al., 2002). Traditionally, the data of inputs and outputs of the different DMUs are assumed to be measured with precision (see, e.g., Cooper et al., 2002; Charnes et al., 1978; Conrad & Strauss, 1983). However, as some authors point out (see, e.g., Cooper et al., 2004, 1996), this is not always possible. The results of DEA models may be sensitive to such variations as a DMU, which is measured as the relative efficiency to other DMUs, may turn inefficient if such random variations are considered. Asgharian et al. (Asgharian et al., 2010) used input relaxation approach to congestion in stochastic data envelopment analysis (SDEA). Furthermore, Khodabakhshi et al., proposed a new model to estimate return to scale (RTS) with fuzzy and stochastic data with chance constrained programming approach (Khodabakhshi et al., 2010).

Stochastic input and output variations and chance constrained programming approach into DEA have been studied by Cooper et al. (1996), and Land et al. (1993).

In this paper, the concept of chance constrained programming with stochastic inputs and outputs is used to extend input relaxation stochastic model to identify congestion of six hospitals of Kermanshah University of Medical Sciences in Iran in the period of time 2008-2012. We, then, obtain a deterministic equivalent to input relaxation model, after that we will show that the deterministic equivalent can be transformed to quadratic programming model that is used to identify congestion input of hospitals. In some peppers, DEA has used to evaluate the relative efficiency hospitals that we mention some of them.

Oliver et al. (2012), has reviewed recent studies comparing the efficiency of German public, private non-profit and private for-profit hospitals. Lee et al. (2008), reformed the hospital service structure to improve the efficiency of urban hospital specialization. Using DEA, this article showed that input variables such as the number of beds, doctors and nurses were related to hospital efficiency. Linna et al. (2006), has compared hospital cost efficiency between Norway and Finland by using DEA Models. Ancarani et al. (2009), has evaluated the impact of managerial and organizational aspects on large Italian Hospital wards’ efficiency using DEA. At last, a list of papers can be named without referring to the details which used DEA with different models to evaluate the relative efficiency hospitals and health care in different countries (Grosskopf & Valdmanis, 1978; Valdmanis, 1992; Kirigia et al., 2002; Linna et al., 2006; Sherman et al., 1984; Morey et al., 1990; Chen et al., 2005; Giokas, 2001).

The remainder of this article is organized as follows: in Section 2, input-oriented CCR, BCC and Input relaxation models were described. In Section 3, we provided an input relaxation model based on the model that was introduced in Section 2. In Section 4, stochastic version of the proposed input relaxation model was developed, and its deterministic equivalent was also obtained. Furthermore, it was shown that the deterministic equivalent of the stochastic model could be converted to a quadratic program. As an empirical example, we applied the model to data of six hospitals of Kermanshah University of Medical Sciences in time period 2009 till 2012. At last, section 5 concluded the paper and presented suggestions for future research.

## 2. Method

### 2.1 Input Relaxation Model

Suppose we have n DMUs which DMUj: j =1; 2; …, n; use m inputs *x_ij_*; i=1,2,…,m to produce outputs, *y_rj_*; r =1,2,…,s. The efficiency of DMUo can be evaluated by the CCR model that has been defined by Banker et al. in 1984 (Banker, 1984) as the following form:


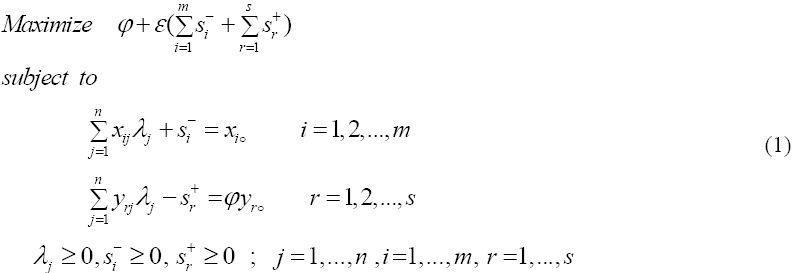


Banker et al. (1984), added the convexity constrained 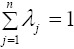 to CCR model (1) to estimate return to scale in DMUs. New model is called output-oriented BCC model as follows:


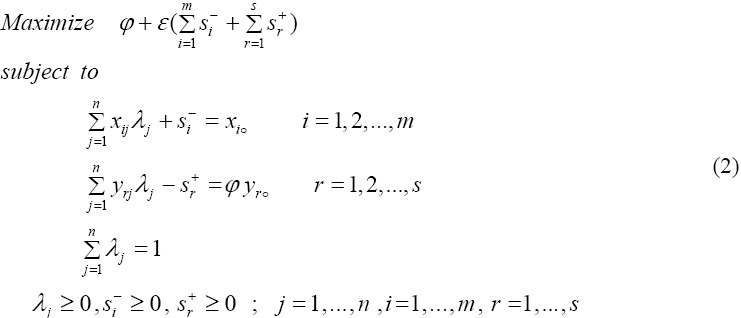


### 2.2 Definition 1

DMUo is efficient in optimal solution model (2) if and only if two conditions are satisfied:

i) φ* = 1;

Ii) *S_i_*^-*^ = *S_r_*^+*^ = ○ for all i and r

Solving models (1) and (2) efficiency and the technical efficiency DMUs, respectively, will be evaluated.

If definition 1 holds, DMUo is efficient according to model (2) otherwise is inefficient. Inefficiency of a DMU causes increasing or decreasing of inputs or outputs, respectively.

### 2.3 The one-Model Approach to Congestion

Cooper et al. (2002) proposed the following model to identifying congestion in inputs that is called one-model approach:


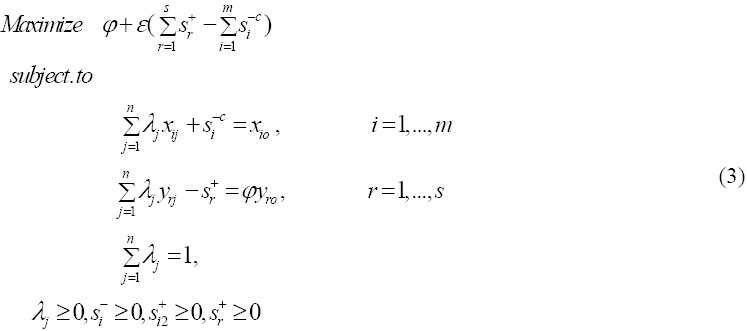


### 2.4 Definition 2

Congestion is present if and only if in an optimal solution (*φ*_o_^*^, *λ*, *S*^+*^, *S*^-c*^ of model (3), at least one of the following two conditions is satisfied:

(i) *φ*_o_^*^ and there is at least one *S_i_*^-c*^ > 1.

(ii) There exists at least one *S_r_*^+*^ and at least one *S_i_*^-c*^ > 1.

The original models, CCR and BCC in DEA only allow the decrease of inputs and increase of outputs in DMUs that are inefficient. Jahanshahloo et al. (Jahanshahloo & Khodabakhshi, 2004), introduced an input relaxation model that allows inputs increase to improve outputs for units which are inefficient. The input relaxation model for improving output


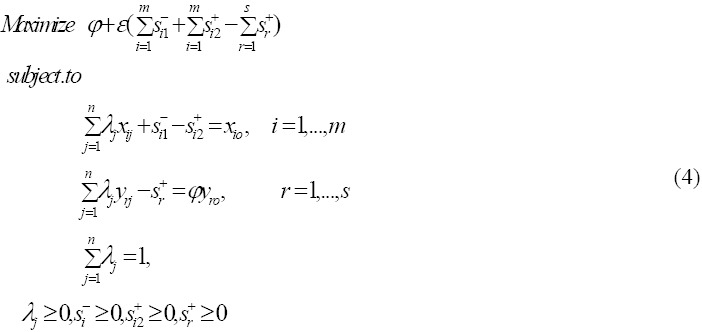


Where *φ* is maximum possible proportional outputs amount that DMUo can produces, and the first and second slacks in the input constraints are slacks for decrement *S*_*i*1_^-^ and increment *S*_*i*1_^+^ of the *i*th input.

### 2.5 Definition

DMUo is efficient for the input relaxation model if the following two conditions are satisfied:

1) φ* = 1

2) *S_r_*^+*^ = *S_i_*^-*^ = *S*_*i*2_^+*^ = 0; ∀ *i*, *r*

### 2.6 Stochastic Input Relaxation Model

Stochastic variations in input and output of DMUs don’t be permitted in ordinary DEA models. While, the evaluating of efficiency DMUs may be sensitive to such variations. A DMU which is efficient relative to other DMUs may turn inefficient if such random variations are considered. The stochastic version of DEA method that has been called stochastic data envelopment analysis (SDEA) is used for planning purposes when inputs or outputs of the DMUs are random variables. Following Cooper et al. (2004) and Khodabakhshi et al. (Khodabakhshi et al., 2010), let *x̃*_*j*_ = (*x̃*_1*j*_,...,*x̃*_*mj*_)^*T*^ and *ỹ*_*j*_ = (*ỹ*_1*j*_,...,*ỹ*_*sj*_)^*T*^ represent random input and output vectors, respectively, and *x*_*j*_ = (*x*_1*j*_,...,*x*_*mj*_)^*T*^, also *y*_*j*_ = (*y*_1*j*_,...,*y*_*sj*_)^*T*^ stand for the corresponding vectors of expected values of input and output for each DMUj; j = 1,2,… n. In other words, these expected values are utilized instead of the observed values in model (1). Let us consider all input and output to be jointly normally distributed in the following chance-constrained stochastic DEA model which is stochastic version of model (4).


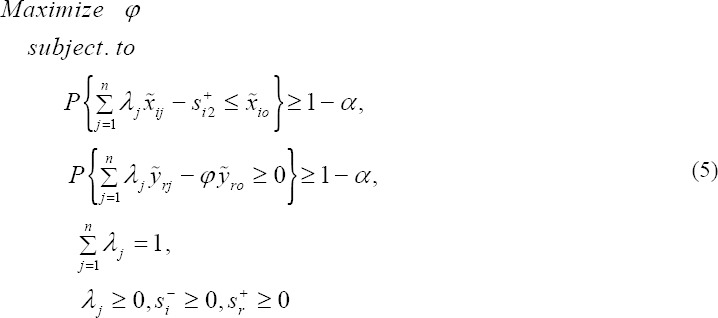


In above model, P means probability and *α* is a predetermined value between 0 and 1.

### 2.7 Deterministic Equivalents

In this section of the article we are going to find a deterministic equivalent for the stochastic model (5) by using normal distribution function. By adding positive *ζ_i_* variable to the *i*th input chance constraint in model (5) we will have:


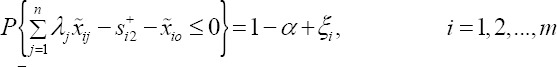


There is a positive number *S*_*i*1_^-^ such that


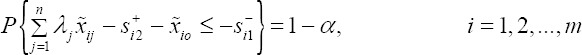


Similarly by adding a positive variable *ζ*_*r*_ to the rth output chance constraint in model (5) we will have:


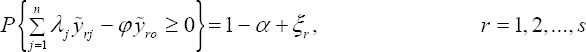


Again, there is a positive number *S*_*r*_^+^ such that


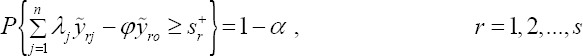


Therefore, we can change the model (5) as follows:


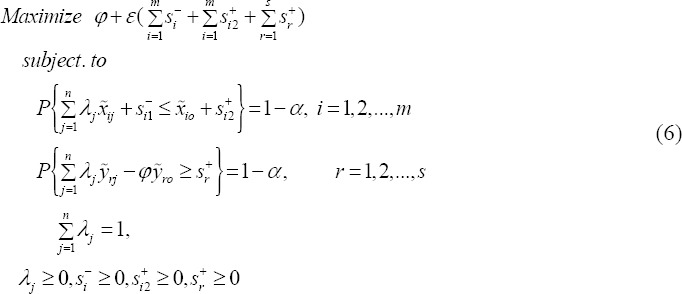


In model (6), for the *i*th input constraint, have


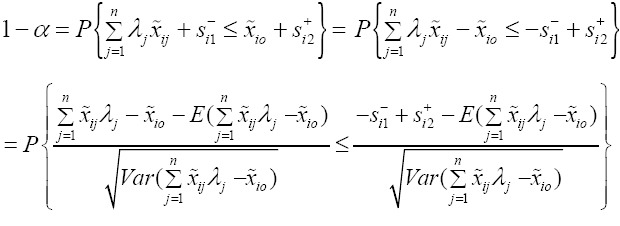


Now, we Let, 
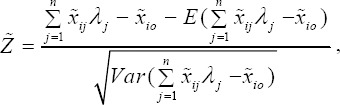
 where *Z̃* is the standard normal random variable (with zero is mean and unit variance). Suppose *ϕ* is the cumulative distribution of the standard normal random *Z̃*, therefore, the inverse of the cumulative distribution *ϕ* exist and is called *ϕ*^-1^.


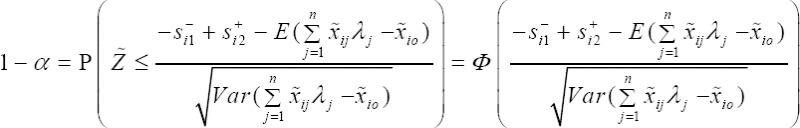


Therefore, from above model we have


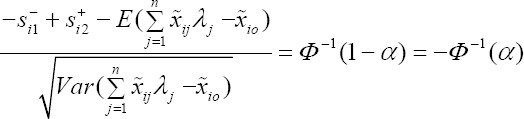


From the above equation, we will have


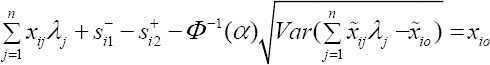


We let, 
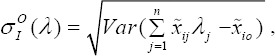
, then


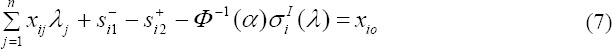


Where


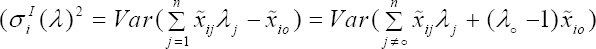



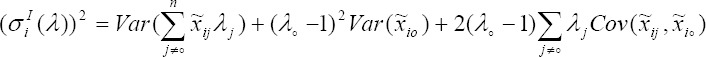



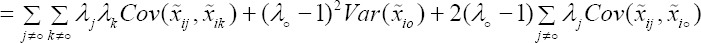


Similarly what we did for input constraints, output constraints in model (6) will be converted as follows:


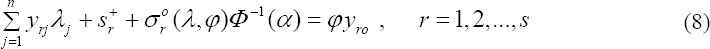


Where


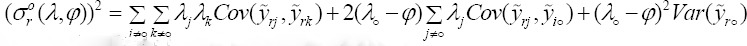
 Therefore, stochastic model (6) has a deterministic equivalent as follows:


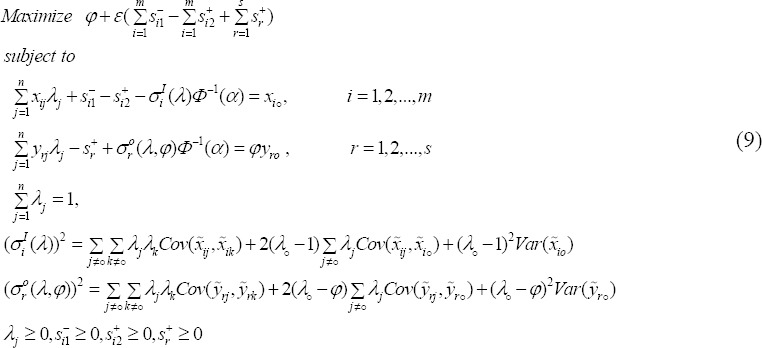


Where *ϕ* is the cumulative distribution function (CDF) of a standard normal random variable and its inverse is *ϕ*^-1^. Following Khodabakhshi et al. (2010), we can show that nonlinear model (9) is a quadratic programming problem. By solving the quadratic program (9) one can obtain the optimal values φ^*^, *S*_*i*1_^-*^, *S*_*i*2_^+*^, and *S*_*r*_^+*^. One of the following three cases should naturally occur for the *i*th input of evaluating DMUo:

(i) Increase, which corresponds to *S*_*i*2_^+*^ > 0.
(ii) Decrease, which corresponds to *S*_*i*1_^-*^ > 0.
(iii) no change, which corresponds to *S*_*i*1_^-*^ = *S*_*i*2_^+*^

### 2.8 Congestion to Stochastic Input Relaxation Model

Now, we can use the input relaxation model to identify and estimate levels of congestion inputs when inputs and outputs aren’t real as follows:


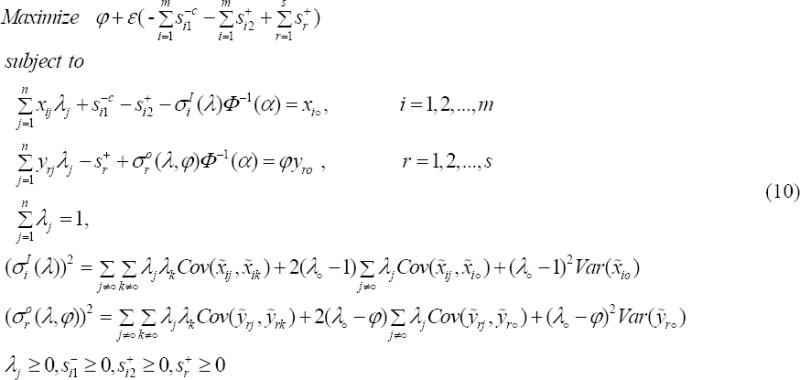


### 2.9 Definition 4

Congestion is present if and only if in an optimal solution *φ*_o_^*^, *λ*, *S*^+*^, *S*^-c*^ of model (10), at least one of the following two conditions is satisfied:

(i) *φ*_o_^*^ > 1 and there is at least one *S_i_*^-c*^ > 1.

(ii) There exists at least one *S_r_*^+*^ and at least one *S_i_*^-c*^ > 1.

## 3. Result

### 3.1 Application

Now, we use the last model to identify congestion and estimate levels in only input Staff with data of six hospitals in the state of Kermanshah, Iran from 2009 to 2012, which is presented in Table 1.

**Table 1:** Data of iranian hospital 2009 to 2012

Year	Hospital	Input	Output	

		I1	O1	O2
2009	H1	326	45332	31846
	H2	948	47207	54787
	H3	904	61802	882174
	H4	542	69738	16284
	H5	3434	145678	165687
	H6	250	76378	8639
2010	H1	413	50961	42657
	H2	909	56323	81997
	H3	818	62468	119426
	H4	488	58986	19712
	H5	3077	150143	204638
	H6	263	74069	12122
2011	H1	425	62664	58984
	H2	871	59961	88618
	H3	743	63184	137839
	H4	483	66745	24001
	H5	3437	162451	269792
	H6	284	113659	11828
2012	H1	469	58448	67872
	H2	777	59284	95926
	H3	757	79847	163634
	H4	486	72575	34997
	H5	3740	160874	319929
	H6	312	100080	30034

To compute the results of the stochastic input relaxation model *α* =0.2 has been chosen. So, from a cumulative normal distribution table, we have *φ*(0.2)=-0.84 and also, the input and outputs variables considered in the present study are as follows:

Input: 1- Staff (I1) Outputs: 1- Outpatient (O1) 2- Revenues (O2)

Then we use stochastic input relaxation model (10) to identify congestion and obtain its measure for data of Table 1. Table 2 shows the results.

**Table 2:** Results of Congestion of iranian hospital with α=0.2

Year	Hospital	For model (10)	Labor changes		Outputs slack of model (2)	

			*s*_11_	*s*_11_	*s*_1_	*s*_2_
2009	H1	3.549	0	3414	0	206915
	H2	3.408	0	2792	0	133220
	H3	1.000	0	0	0	0
	H4	2.307	0	3198	0	282360
	H5	1.104	0	306	0	136960
	H6	2.106	0	3490	0	301730
2010	H1	3.157	0	3327	0	185270
	H2	2.856	0	2831	0	85723
	H3	2.575	0	2922	0	12371
	H4	2.727	0	3252	0	266170
	H5	1.412	2173	0	0	797940
	H6	2.172	0	3477	0	293600
2011	H1	2.567	0	3315	0	168500
	H2	2.683	0	0	0	82168
	H3	2.484	0	2884	0	0
	H4	2.410	0	3257	0	262080
	H5	1.380	2533	0	0	779540
	H6	1.415	0	3456	0	303190
2012	H1	2.752	0	3271	0	133120
	H2	2.714	0	2963	0	59623
	H3	1.999	0	2947	0	0
	H4	2.217	0	3254	0	242350
	H5	1.141	2836	0	0	759270
	H6	1.607	0	3428	0	271650

Results of the deterministic equivalent of the stochastic input relaxation model, model (10), are presented in Table 2. Columns 3, 4-5 and 5-6 of the Table represent score efficiency, labor changes, outputs slack of hospitals for stochastic input relaxation model, model (10), respectively. Note that an efficiency score equal 1 implies that the DMU is efficient and scores greater than 1 imply that the DMUs are inefficient.

From computational results presented in column 3 of Table 2, using Definition 1, H3 with efficiency score *φ*_o_* is efficient and the rest of the hospitals are inefficient. The worst hospital is H1 with efficiency score *φ*_o_* = 3.549. This hospital can produce 3.549 times of its current outputs, i.e., 3.549*(45332, 31864) = (160883, 113022). Based on the numerical results presented in column 3 of Table 2, using Definition 2, it is observed that the fifth hospital, H5, at years 2010, 2011 and 2012 is inefficient and congested and the value of congestion at these years is 2173, 2533 and 2836, respectively. This hospital has worked with 2173, 2533 and 2173 additional personal at years 2010, 2011 and 2012, respectively. Decreasing these numbers of personal in these hospitals, their outputs will increase and consequently the application of hospital may improve. The rest of hospitals have not congestion in personal input.

## 4. Conclusion

This paper discussed congestion in stochastic data envelopment analysis with input relaxation model. The deterministic equivalent of the stochastic version proposed by the model was converted to a nonlinear (quadratic) programming. As an application example, the proposed approach was also applied to data of Iranian hospitals. Computational results from stochastic input relaxation model showed that hospital H5 was inefficient during 2009-2012 and included staffs congestion during the last three years of the study. Finally, developing the proposed model in fuzzy, data envelopment analysis is suggested for further research.
